# Comparative analysis of completeness of death registration, adult mortality and life expectancy at birth in Brazil at the subnational level

**DOI:** 10.1186/s12963-020-00213-4

**Published:** 2020-09-30

**Authors:** Bernardo L Queiroz, Marcos R. Gonzaga, Ana M. N. Vasconcelos, Bruno T. Lopes, Daisy M. X. Abreu

**Affiliations:** 1 Graduate Program in Demography, Universidade Federal de Minas Gerais, Av. Antonio Carlos, Belo Horizonte, 6627 Brazil; 2 Department of Demography and Actuarial Science, Universidade Federal do Rio Grande do Norte, Natal, Brazil; 3 Department of Statistics, Universidade de Brasilia, Brasilia, Brazil; 4 Program in Actuarial Science, Instituto de Ciências Exatas, Universidade Federal de Minas Gerais, Belo Horizonte, Brazil; 5 School of Medicine, Universidade Federal de Minas Gerais, Belo Horizonte, Brazil

**Keywords:** Completeness of death registration, Demographic methods, Mortality estimates, Subnational analysis, Brazil

## Abstract

**Background:**

Estimates of completeness of death registration are crucial to produce estimates of life tables and population projections and to estimate the burden of disease. They are an important step in assessing the quality of data. In the case of subnational data analysis in Brazil, it is important to consider spatial and temporal variation in the quality of mortality data. There are two main sources of data quality evaluation in Brazil, but there are few comparative studies and how they evolve over time. The aim of the paper is to compare and discuss alternative estimates of completeness of death registration, adult mortality (45q15) and life expectancy estimates produced by the National Statistics Office (IBGE), Institute for Health Metrics and Evaluation (IHME), and estimates presented in Queiroz et al. (2017) and Schmertmann and Gonzaga (2018), for 1980 and 2010.

**Methods:**

We provide a descriptive and comparative analysis of aforementioned estimates from four (4) sources of estimates at subnational level (26 states and one Federal District) in Brazil from two different points in time.

**Results:**

We found significant differences in estimates that affect both levels and trends of completeness of adult mortality in Brazil and states. IHME and Queiroz et al. (2017) estimates converge by 2010, but there are large differences when compared to estimates from the National Statistics Office (IBGE). Larger differences are observed for less developed states. We have showed that the quality of mortality data in Brazil has improved steadily overtime, but with large regional variations. However, we have observed that IBGE estimates show the lowest levels of completeness for the Northern of the country compared to other estimates. Choice of methods and approaches might lead to very unexpected results.

**Conclusion:**

We produced a detailed comparative analysis of estimates of completeness of death registration from different sources and discuss the main results and possible explanations for these differences. We have also showed that new improved methods are still needed to study adult mortality in less developed countries and at a subnational level. More comparative studies are important in order to improve quality of estimates in Brazil.

## Background

Adequate knowledge of mortality levels in a country and its regions is necessary for efficient planning of public policies, especially health and social security, to better understand the impacts of regional (economic and social) differentials and also to carry out work of research in different areas of knowledge, specifically demography, economics and public health. However, studies on mortality in developing countries, such as Brazil, have been limited by the lack of a better quality in data, problems with completeness of death registration and the quality of population information [1–5]. In general, the problems encountered in the data for the country are aggravated when dealing with population subgroups and/or at a subnational level [6, 7]. Even in developed countries, there are differences in the quality of vital statistics at subnational level (and population sub-groups). These differences can be attributed mainly to the degree of economic development of each region [8, 9].

Currently in Brazil, two main sources of mortality estimates are used in public policy formulation and monitoring: (1) IBGE (Instituto Brasileiro de Geografia e Estatística—the Brazilian statistics agency) produces officials mortality estimates used for population forecasts, social security policies and general planning, and (2) IHME (Institute of Health and Metrics Evaluation) provides mortality estimates from the Global Burden of Disease study (GBD) used for public health policies at national and subnational levels. Even though there are great differences between these estimates, few comparative analyses have been made. Thus, it is relevant to undertake a systematic comparison and contrast the results, providing some guidance on how methods and data might explain the observed differences.

In this paper, we focus on comparing adult mortality estimates—_45_q_15_, and life expectancy at birth in Brazil over 1980 and 2010—and estimates of completeness of death registration at subnational level. In order to compare these two main sources, we add to the analysis other mortality estimates from two independent studies: (1) Queiroz et al. [9]—DDM-R—which provide estimates of completeness of death registration for two periods 1980/1991 and 2000/2010, which is fundamental in assessing the quality of data and for the correction of adult mortality at subnational level, and (2) Schmertmann and Gonzaga [6], based on Bayesian models, produced estimates of adult mortality, life expectancy at birth and probabilities of dying by single age at subnational level for 2010.

The main contribution of this paper is to highlight differences in adult mortality estimates and life expectancy at birth, currently used to support public policy decisions in Brazil and regions. The discrepancies must be explained, and it is important to have transparency and replicability when producing mortality estimates for countries with limited data, mainly at subnational level which have huge implications for public health planning.

## Data and methods

### General overview of data and methods

We provide a descriptive and comparative analysis of different sources of estimates in Brazil at the subnational levels, at two different points in time. Our analysis focused on comparison of the estimated completeness of death registration produced by IBGE and IHME to those produced by IBGE and DDM-R [9] and, for 2010, to estimates from the Bayesian model [6].

We focused on completeness of reporting of deaths, adult mortality and life expectancy at birth. We first focused on the quality of mortality data, which is measured by the completeness of reporting of deaths. From the estimates of completeness, it is possible to adjust mortality data and obtain estimates of adult mortality (45q15) and life expectancy at birth. It is important to notice that we did not compare how each agency estimate infant, child and old-age mortality, and these estimates impact on the estimated levels of life expectancy. However, we believe that it is important to evaluate differences in life expectancy at birth for their impact on other studies. We concentrated our analysis on the intercensal periods 1980–1991 and 2000–2010. The use of the two extreme intercensal allows one to capture important variations over time and place and also highlight the main differences in the estimates produced for quality of death registration and mortality in Brazil.

To compare different estimates over time, we used the root square mean error (RMSE) as our measure of convergence in data quality and adult mortality estimates over-time. RMSE is a measure of the difference between values predicted by a model and the values observed and also provides the information of convergence or divergence across the different estimates investigated.

### Death distribution methods

In general, the studies use the death distribution methods (DDM) to perform the analysis of mortality data quality and mortality estimates. We present an overview of each method and highlight the main points of each one since how each one approaches the methods might affect the final estimates. Table 1 provides a summary of all methods and a brief description of the application by IBGE, IHME and DDM-R. DDM derives from the population dynamics equation and compares the distribution of deaths by age with the age distribution of the population and provides the mortality age profile for a defined period [13]. There are three main methods related to DDM: general growth balance (GGB) proposed by Hill [13], synthetic extinct generation (SEG) proposed by Bennett and Horiuchi [14] and adjusted synthetic extinct generations, proposed Benneth and Horicuhi [14] and presented in Hill et al. [15]. The methods have very strong assumptions [16, 17]: (1) population is closed to migration or observed very few migration flows, although there are some methodological alternatives to that when data is available or by adjusting the method [16, 17]; (2) the degree of completeness of reporting of deaths is constant by age; (3) there are no differences in completeness of the census data (population) by age and in each census; and (4) there are no errors in the declaration of ages of the living or the dead [10–13].

**Table 1:** Overview of differences in modelling approach used by IHME, IBGE and DDM-R for estimating completeness of death registration. Albuquerque and Senna [10], IHME [11] and IBGE [12]

Method	Overview
GGB	• Requirements: 2 population age structure, death counts by age • Assumptions: no age misreporting, completeness constant by age, closed population or very small migration flows • Basic idea: balancing equation, death rates = birth rates − growth rates • Model as a regression model. Intercept gives the relative coverage from the two-population age structure, slope gives an estimate of relative completeness of death registration
SEG	• Requirements: 2 population age structure, death counts by age • Assumptions: no age misreporting, completeness constant by age, closed population or very small migration flows, coverage constant across population counts • If we follow a cohort, number of people aged 0 is equals to the number of deaths aged 0 in year *t*, plus deaths aged 1 in year *t* + 1 and so on. Use age-specific growth rates and current deaths to estimate future cohort deaths • Completeness is obtained by comparing observed population to population obtained from death counts
SEG-adj	• Requirements: 2 population age structure, death counts by age • Basic idea: uses GGB to obtain relative coverage from the two-population age structure. Adjust population so they have the same level of completeness. Apply SEG after this adjustment.
DDM-R	• Provides estimates of the 3 basic death distribution methods • Age segments used to obtain intercept and slope in GGB are given by testing different adjustment and selecting the best fit using RMSE • Same age group is used to estimate SEG and SEG-adj
IBGE	• In 1980–1991 and 1991–2000 uses the original version of the Growth Balance that assumes stable population • 2000–2010 used GGB, but does a top-down adjustment to the country level to correct subnational level
IHME	• Documentation suggests that uses all three versions of DDM • SEG 55–80, GGB 40–70, and GGBSEG 50–70 are the best methods that can be currently used to estimate relative completeness of death registration • Documentation is not clear on which age range is used to obtain estimates for Brazil and subnational level.

The general growth balance (GGB) method is derived from the basic demographic equilibrium equation, which defines population growth rate as the difference between the population’s input rate and the population’s output rate. This relationship [13] also occurs for any age interval with open interval *x* +, and in a closed population (or one with small migration flows), entries occur as birthdays at ages *x*. Thus, the difference between the input rate *x* + and the population growth rate *x* + produces a residual estimate of the mortality rate *x* + [13, 15]. If the residual mortality estimate can be estimated from two population censuses and compared with a direct mortality estimate using the demographic census or death counts enumeration, the completeness of death registration can be estimated [13, 15].

Equation (1) presents a formalization of the GGB method:

1 $$ \frac{N(x)}{N\left(x+\right)}-r\left(x+\right)=\frac{1}{t}\ast \mathit{\ln}\left(\frac{k_1}{k_2}\right)+\frac{{\left({k}_1\ast {k}_2\right)}^{1/2}}{C}\ast \left(\frac{D\left(x+\right)}{N\left(x+\right)}\right) $$

where *N*(*x*) is the number of persons who reach the exact age *x* in the period, ***N***(*x*+) is the number of persons of at least age *x*, *r*(*x*+) is the population growth rate for ages *x* and higher, the ratio of *k*_1_ and *k*_2_ is the relative coverage of the population enumeration in the two censuses, *C* is the degree of completeness of death records over the period, *D*’(*x*+) is the observed number of deaths of people aged *x* or higher, and *t* is the length of the intercensal interval. Thus, the input rate minus the growth rate has a linear relationship with the mortality rate. From this equation, one can calculate the degree of completeness of death records (C) over a period and the relative coverage of the population enumerated in the two censuses (*k*_1_/*k*_2_). It should be noted that the method compares the age distribution of deaths (mean in the intercensal period) with the population change between censuses, i.e. specifically, the estimate refers to the coverage of the record between the censuses and not to the final or initial period of study.

Bennett and Horiuchi [14] suggest an alternative way to use census information and a distribution of deaths by age to evaluate the completeness of death counts registration. Population growth rates are used to expand the observed distribution of death by age to a stationary population or to a life table distribution. Because on a life table deaths over age *a* are equal to the life table population at the exact age *a* (since all die), the ratio between expanded deaths over age *a* and an estimate of population over age *a* obtained from the census enumerations give estimates of the completeness of death registration in relation to the census coverage. The main difference between the two models is that Bennett-Horiuchi can be applied to nonstable populations.

That is, the extinct generation method (SEG) uses age-specific growth rates to convert an age distribution of deaths into an age distribution of a population. Age-specific growth rates are used to adjust the number of deaths from the stationary population to a nonstable population. The sum of deaths over age *x* provides the population estimate of age *x*. The degree of completeness of the death counts is given by the ratio of the population estimated by death registration over age *x* to the observed population at age *x*.

Equation (2) gives the mathematical formalization of the SEG method:

2 $$ \hat{c}(a)=\frac{\mathrm{N}\hat{\Big(}a\Big)}{{\mathrm{N}}^0(a)}=\frac{\int_{\mathrm{x}=\mathrm{a}}^{\overline{w}}\mathrm{D}\left(\mathrm{x}\right){\mathrm{e}}^{\int_{\mathrm{a}}^{\mathrm{x}}\mathrm{r}{\left(\mathrm{ydy}\right)}_{\mathrm{dx}}}}{{\mathrm{N}}^0(a)} $$

where *N’x* is the number of people who reach the exact age *x* in a population with growth rate *r* and *Dx* is the number of deaths at age *x*. In this case, the estimate of completeness of death registration, $\overset{\wedge }{c}(a),$ is given by the ratio between the estimated number of people aged *x*, *N’x*, to the observed number of people aged *x*, *Nx*.

Both methods offer qualitative measures to assess data quality and validate results. In the GGB method, the quality analysis is made using the observed mortality rate and the estimated residual mortality rate. If this relationship is very close to a straight line (model fit), the quality of the information can be considered good [15, 18, 19]. If the GGB diagnostic plot is far from the adjustment line, this will indicate problems and limitations in age declaration and migration effects. The diagnostic plot of the SEG method shows the degree of coverage to be constant over the age range. A change in the slope of the line across age groups indicates possible problems in varying coverage of demographic censuses, problems in declaring the age of the living and the dead or s variation in the quality of registration/enumeration of deaths by age group [20, 21].

Hill et al. [13] and Murray et al. [19] based on a series of simulation estimates argue that the combination of both methods produce more robust results. Their main argument is that the GGB has a good ability in estimating the quality of the census relative to the other and would allow for the best fit and that SEG is less sensitive to variation in data quality. Dorrington and Timaeus [22] show a comparative analysis of different DDM methods and argue that the SEG + delta, which considers different coverage among censuses would work better in different scenarios. The main caveat of the method is that they assume closed population or very small migration flows for better use of estimates. Although there are methodologies in the literature that allow us to deal with this problem [16, 17], they demand the existence of good quality data on migration or the use of migration models. Thus, when working with aggregated country data and regional data, it is important to consider the possible effects of migratory flows on the assessment estimates of data quality. Another alternative applied in regional studies using death distribution methods is to use an age range to estimate the degree of coverage that suffers little or no influence from migratory flows, instead of the methodology proposed by others [16, 17].

Based on estimates of completeness, all sources also provide information on adult mortality, _45_q_15_, as the probability of a 15-year-old dying between ages 15 and 60. We use adult mortality estimates because they are some of the most important sources of information IBGE and IHME use to obtain complete life tables using relational models. Thus, adult mortality is not considered here as an indicator of the accuracy of estimates, but to discuss how different estimates by each author might lead to very different life table estimates. Adult mortality is a simple measure and allows for comparison between studies. We consider that the entry into adulthood occurs at age of 15 years and at that age, there is the inflection point in which the declining of childhood mortality risks is replaced by increased mortality risks for young adults and adults. In addition, this measure covers a substantive age interval—up to the age of 60—and avoids problems inherent in estimates of mortality at more advanced ages.

#### IHME—Global Burden of Disease

The method used by IHME, based on GDB 2017, for the estimates we analysed in this study is available at https://vizhub.healthdata.org/mortality/ [23]. A more detailed description of the method used to estimate life tables is available elsewhere [24, 25]. In summary, death reporting from vital registration and censuses were evaluated for completeness using “improved death distribution methods”. However, it is not very clear from the description of the method and the material available what “improvement” they made to the death distribution methods—the paper mentions that these were developed based on simulations from Murray et al. [19], but does not specify age ranges used or what the procedures were when completeness was above 100%. We were also not able to identify the codes and programme used to estimate completeness for Brazil and regions. In addition, they applied an improved sibling survival method to survey data on sibling survival modules to correct for survivor bias, zero-survivor bias and recall bias. Estimates of under-5 and adult mortality were generated using a combination of spatio-temporal and Gaussian Process regressions.

#### IBGE

IBGE has been using different methods and data to produce mortality estimates for state levels since 1980 [10, 12]. For 1980–1991, 1991–2000 and 2000–2010 intercensal periods, despite of the limited applicability of the method to nonstable populations and in the context of large migration flows between states in Brazil, IBGE used the growth balance method [13, 15] to estimate the completeness of death registration in most states. However, there are some specificities in their application. For females in Northeast region in 1980 and in North and Northeast regions in 2000, IBGE used other methods [10]. Based on expert opinions, a reduction factor was applied to adjust the deaths for under-reporting in each year in order to produce the best estimates of the adult and elderly population in relation to the young and young adult deaths [10]. For infant mortality, IBGE used indirect demographic methods [20] but this goes beyond the scope of this paper since we are mostly interested in estimating adult mortality and completeness of death registration for adult ages.

In 2010, IBGE made some changes on the methodology to estimate life tables for Brazilian states. For Southern states plus São Paulo, Rio de Janeiro and Distrito Federal, death records were used without any correction for under-reporting. In all other states, the general growth balance method [13] was used. Top-down estimation procedures were applied in order to make sure that the sum of deaths between states was equal to national total estimated deaths [12]^1^.

#### Queiroz et al. [9]—DDM-R

Queiroz and collaborators [9] evaluate the completeness of reported deaths using death distribution methods. They use the R–package (DDM), developed by Everton Lima, Tim Riffe and Bernardo Queiroz, focussing on inter-censuses years (1980–1991, 1991–2000, 2000–2010). They build on previous work by Agostinho and Queiroz [1, 2]. Population data, by age and sex, are obtained directly from the National Statistics Office (IBGE) (www.ibge.gov.br) and mortality data are obtained from the Mortality Information System (MIS) of Ministry of Health (available at www2.datasus.gov.br). MIS provides information on deaths by age, sex and causes of death at local levels since 1979. MIS data comes from death certificate where causes of death are registered according to the international form recommended by the International Classification of Diseases. Data on causes of death are coded using the Revision of the International Classification of Diseases (ICD) codes (9th, from 1980 to 1995, and 10th, from 1996 onwards).

#### Bayesian model

Schmertmann and Gonzaga [6] do not use directly DDM in their estimates. They combine a relational model for mortality schedules with probabilistic prior information on completeness of death registration obtained from several studies, and from field audits done by public health experts [26].

### Comparison of estimates

We use the root mean square error (RMSE), also known as root mean square deviation, as a measure of convergence in data quality and adult mortality. The RMSE is calculated as follows:

$$ \mathrm{RMSE}=\sqrt{\sum \limits_{i=1}^N\frac{{\left({x}_i-\underset{\_}{x}\right)}^2}{N}} $$

where *x*_*i*_ é the estimated completeness of death registration from the method *i* and $\underset{\_}{x}$ is the average between estimates in each period and considering level of completeness and adult mortality for all sets (1980/1991 and 2000/2010); *N* é is the number of methods compared in the paper.

RMSE can be interpreted as the variability measure of each estimate in relation to the average estimate between estimates from each method in that particular period. A RMSE decreasing between 1980/1991 and 2000/2010 indicates that there is a convergence across estimates over time.

## Results

**Fig. 1:**
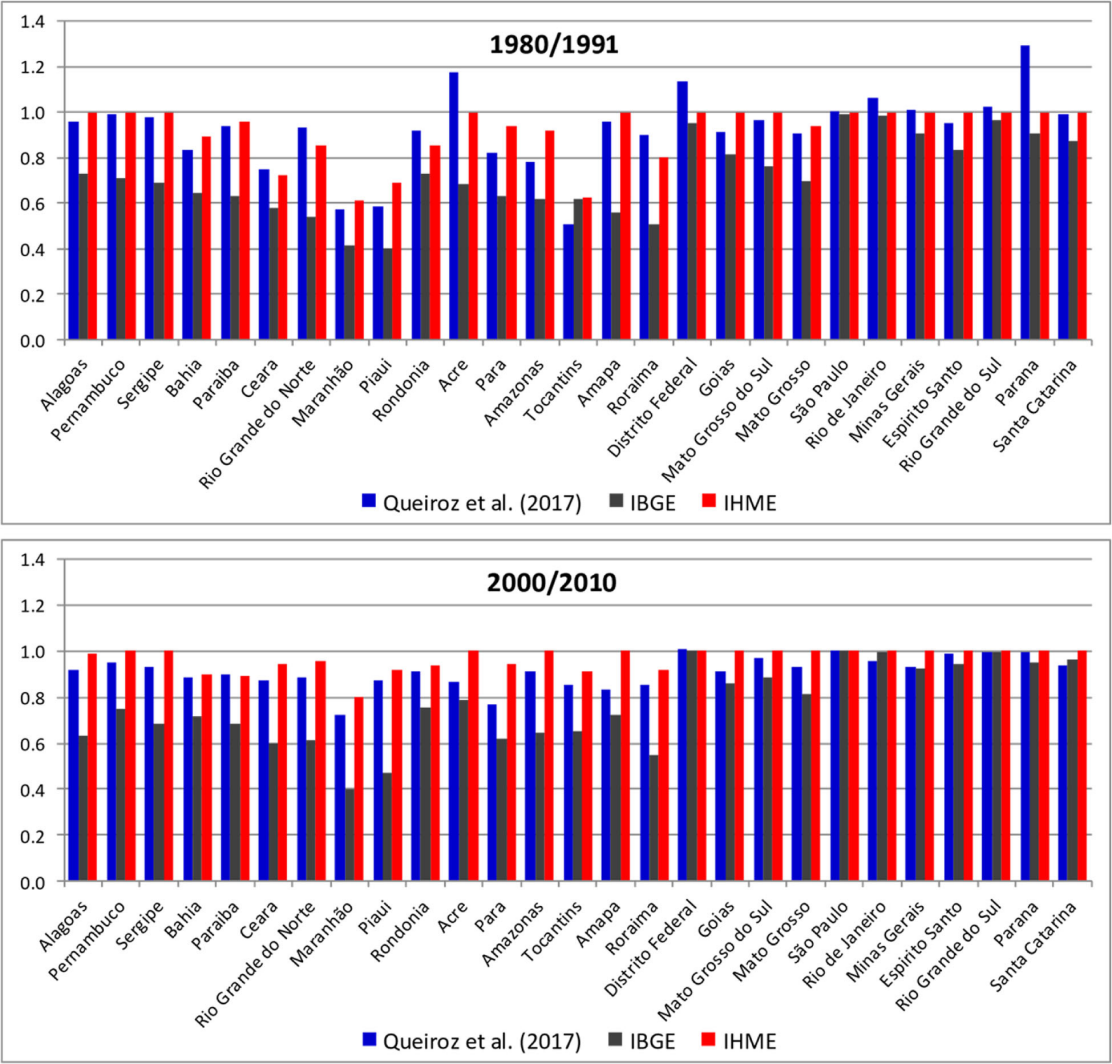
Completeness of death registration, Brazil—Queiroz et al. [9], IHME and IBGE, 1980/1991 and 2000/2010. Source: Queiroz et al. [9], IHME [11] and IBGE [12]

Figure 1 shows estimates of completeness of death registration for males and females by states in the two intercensal periods. We show in Supplementary Table 1 the estimates of completeness and adult mortality for each method and year. Some interesting and important patterns emerge from this analysis: (a) IBGE has the lowest estimates of completeness for states in the northeast during the whole period of analysis, and there was little improvement in data quality from 1980 to 2010 for the Northeast states. For instance, Maranhão has completeness estimated as below 80% in 2000–2010 period; (b) IHME presents a high level of completeness for almost all states since 1980, even for states with very low levels of social and economic development; (c) there is a pattern of slight underestimation between IHME and DDM-R, that is, estimates by DDM-R are normally lower than those produced by IHME, and the states that had the largest discrepancies were the less developed ones, such as Acre, Amazonas and Maranhão (Fig. 1); and (d) estimates of completeness in 2000/2010 for IHME and DDM-R are very similar for males and females and for all states, but they are quite different from estimates produced by IBGE.

**Fig. 2:**
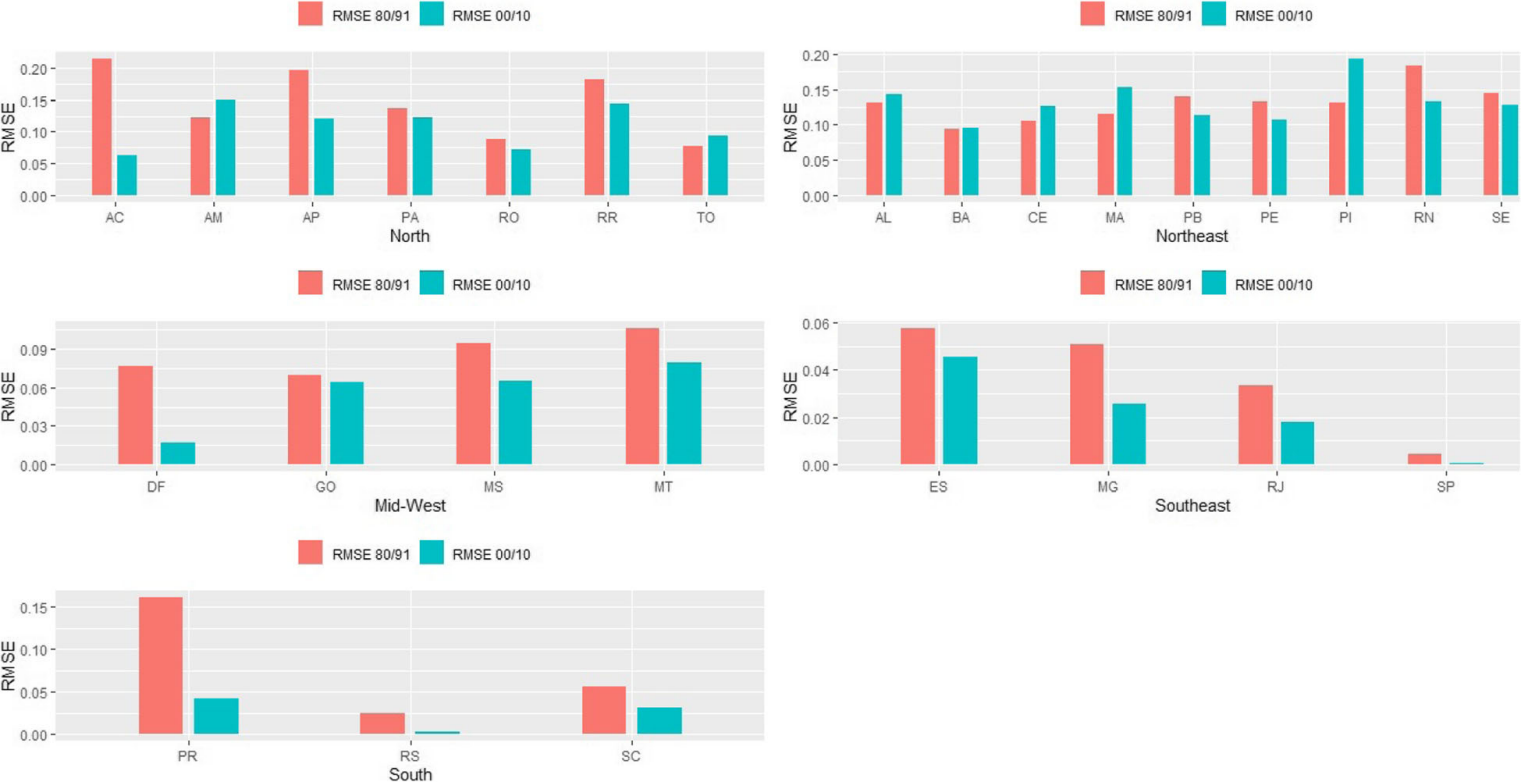
Measure of completeness of death registration convergence—RMSE, by states in Brazil; Males 1980/1991 and 2000/2010. Source: Queiroz et al. [9], IHME [11] and IBGE [12]

In order to evaluate the process of convergence across estimates over period of analysis, we show estimates of RMSE, by state level, for the two intercensal periods and three sets of estimates. For the whole country, the measure of convergence declined from 1980–1991 to 2000–2010 from 0.101 to 0.079 indicating that estimates from the three sources are much like each other in the recent period. Figure 2 shows the results for males and females and each Brazilian state. The results indicate that, on the one hand, the estimates by each different agency converge for the states of South, Southeast (highest level of convergence) and Northeast regions. On the other hand, convergence index for Northern states do not show any changes over time and there is an increase in the divergence for states in the Midwest. We also estimated RMSE for each period of time and for each source of information separately. Completeness of death registration and adult mortality converge from 1980–1991 to 2000–2010 using estimates from DDM-R and IHME. This indicates that overtime quality of vital records and adult mortality are converging to the same level. However, we do not observe any convergence using IBGE data. This is explained because completeness of death registration in the states of Northeast and North did not improve overtime whereas the rest of the country improved rapidly for IBGE estimates, leading to a continuous divergence in completeness and adult mortality.

We also observed another relevant matter when comparing IHME and IBGE to DDM-R. The estimates, from Fig. 1, show that IHME and IBGE do not have estimates of completeness of death registration that exceeds 100%, whereas DDM-R showed results above 100%. Completeness above 100% is possible using DDM, since completeness is estimated relative to the estimate of population, rather than to the absolute level of death registration. That is, if there is a large variation in the quality of population counts (quality of census data varies across country) and deaths are better registered, one can obtain such values [27]. This indicates that IHME and IBGE estimates might be assuming that the impreciseness of the vital record can only be related to the deaths that were not recorded. Unfortunately, we did not have access to their working code or spreadsheets to better understand the complete approach they follow to obtain their results. Situations with poor data might produce estimates of completeness above 100% indicating that underlying data is poor and not that registration of deaths are complete. One additional issue is that there might happen to be very high levels of completeness of death registration and population undercount in the censuses. Also, estimates above 100% might indicate that the methods applied to those contexts are not robust and the strong the assumptions one has to make do not hold. The problem may be even worse, since they might have used a combination of both issues. This is a major point for future discussion and research.

### Comparison of adult mortality estimates

Table 2 shows estimates of adult mortality for 1980–1991 and 2000–2010 for IBGE, IHME and DDM-R. We focus the analysis on estimates of males for the same two periods. The results for females show a similar pattern over the periods. In relation to the estimates from IHME compared to DDM-R, we observed the greatest differences in the 1980–1991 period and a convergence in the more recent period (2000–2010). The main differences in the adult mortality probability (45q15) between the National Statistics Office (15, 17) and others (IHME and DDM-R) are from the period 1980/1991. IBGE estimates of adult mortality were much higher than others in most Brazilians states during this period for males, the behaviour of female estimates followed the same pattern in all comparisons.

**Table 2:** Males adult mortality probabilities, 45q15, males, Brazil—DDM-R, IHME, and IBGE, 1980/1991 and 2000/2010. Source: Queiroz et al. [9], IHME [11] and IBGE [12]

State and region	Queiroz 80/91	IHME 80/91	IBGE 80/91	Queiroz 00/10	IHME 00/10	IBGE 00/10	Bayesian 2010	MS 2010
Northeast								
Alagoas	0.2630	0.22	0.3	0.245	0.242	0.296	0.284	0.265
Bahia	0.2206	0.174	0.323	0.212	0.206	0.251	0.236	0.211
Ceara	0.1720	0.197	0.309	0.207	0.183	0.242	0.216	0.195
Maranhão	0.2129	0.246	0.291	0.193	0.183	0.293	0.221	0.176
Paraiba	0.2084	0.195	0.317	0.217	0.220	0.262	0.235	0.219
Pernambuco	0.2627	0.25	0.356	0.256	0.219	0.263	0.251	0.235
Piaui	0.1741	0.254	0.325	0.183	0.166	0.272	0.200	0.177
Rio Grande do Norte	0.1635	0.237	0.272	0.183	0.171	0.207	0.209	0.186
Sergipe	0.2244	0.191	0.302	0.226	0.205	0.258	0.235	0.216
North								
Acre	0.2163	0.253	0.29	0.229	0.179	0.240	0.209	0.189
Amapa	0.1964	0.197	0.301	0.218	0.165	0.209	0.208	0.179
Amazonas	0.2574	0.195	0.326	0.196	0.172	248,000	0.212	0.182
Para	0.2242	0.225	0.279	0.227	0.183	0.249	0.240	0.192
Rondonia	0.2768	0.32	0.35	0.228	0.197	0.243	0.225	0.200
Roraima	0.2213	0.258	0.342	0.241	0.192	0.271	0.213	0.185
Tocantins	0.2058	0.191	0.305	0.199	0.179	0.234	0.220	0.190
Mid-west								
Distrito Federal	0.2119	0.189	0.1653	0.191	0.170	0.171	0.181	0.176
Goias	0.2542	0.242	0.283	0.226	196,000	0.211	0.226	0.209
Mato Grosso	0.2181	0.171	0.291	0.229	0.198	0.218	0.226	0.210
Mato Grosso do Sul	0.2314	0.319	0.297	0.220	0.200	0.205	0.215	0.213
Southeast								
Espirito Santo	0.2669	0.214	0.255	0.233	0.214	0.200	0.231	0.229
Minas Gerais	0.2548	0.243	0.246	0.213	0.191	0.190	0.213	0.201
Rio de Janeiro	0.2990	0.247	0.313	0.259	0.224	0.211	0.239	0.234
São Paulo	0.2709	0.28	0.291	0.224	0.190	0.178	0.199	0.200
Southeast								
Parana	0.1886	0.269	0.307	0.214	0.204	0.190	0.218	0.217
Rio Grande do Sul	0.2480	0.222	0.319	0.208	0.189	0.177	0.201	0.200
Santa Catarina	0.2238	0.225	0.333	0.197	0.172	0.158	0.185	0.183

The estimates are converging with the ones produced by the IBGE in the 2000/2010 period, but we still notice a pattern of slight overestimation of adult mortality for IBGE compared to IHME and DDM-R. One thing that should be observed when considering the estimates from both sources is the fact that the most developed states, like São Paulo and Rio de Janeiro, are usually the ones that have both estimates going in the same direction; meanwhile, some less developed states still have a large discrepancy between estimates from DDM-R and IHME and the ones produced by IBGE.

### Evaluating differences in 2010

**Fig. 3:**
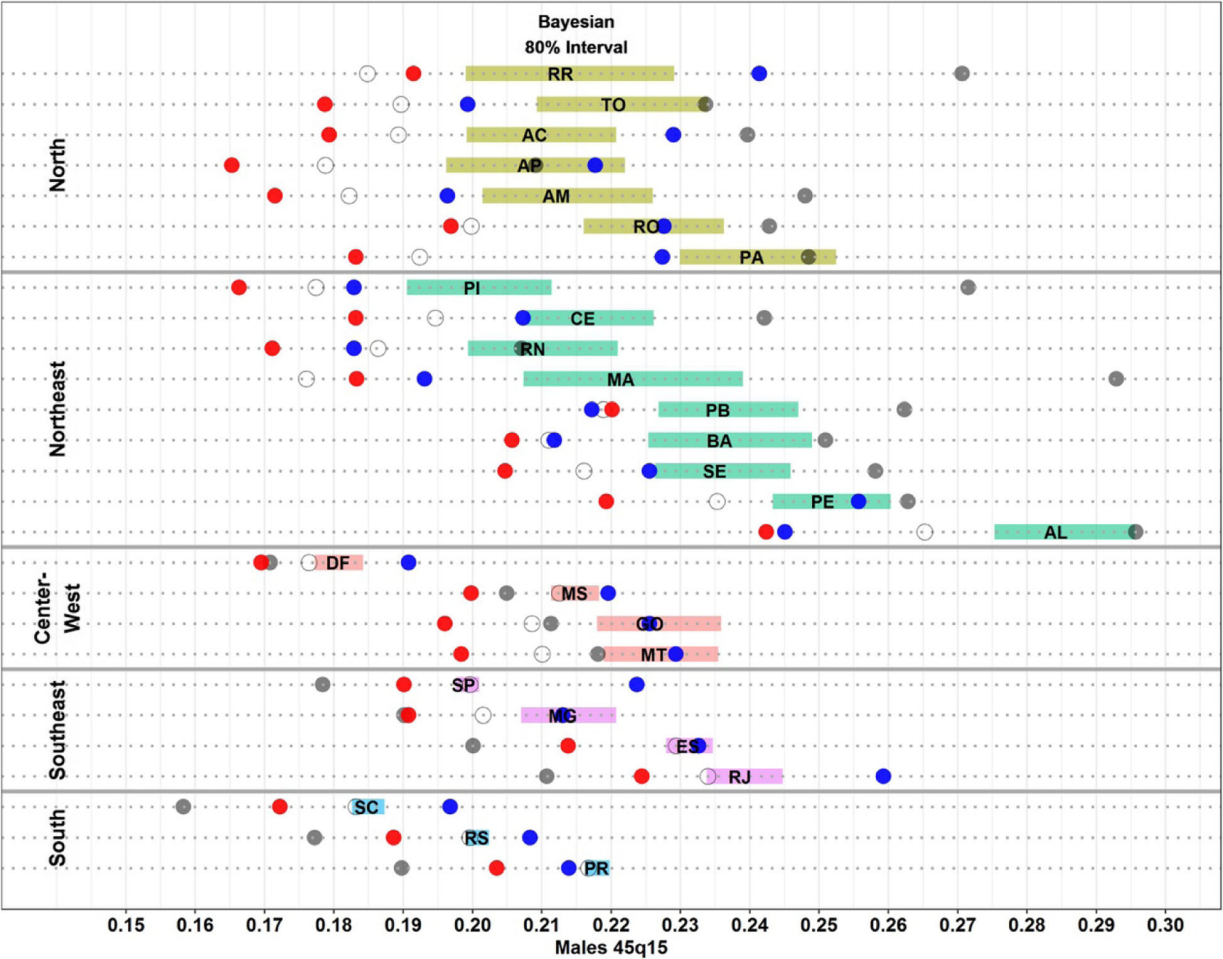
Males adult mortality probabilities (45q15), 2010—Bayesian estimate from Schmertmann and Gonzaga (2018), Queiroz et al. (2017), IHME, IBGE and MIS (Ministry of Health). Source: Schmertmann and Gonzaga [6], Queiroz et al. [9], IHME [11] and IBGE [12]. Note: Shaded bars in different colours represent 80% posterior uncertainty interval for males adult mortality probability (_45_q_15_) from Bayesian model; state abbreviations appear at posterior median for _45_q_15_; red solid dot represent IHME estimates, grey solid dot represent IBGE estimates, blue solid dot represent Queiroz et al (2017) estimates and open circles represent unadjusted estimates from deaths registered by the Mortality Information System (MIS)

In order to perform a more detailed analysis of those estimates, we now focus on results for 2010 for adult mortality. The results show that for the North and Northeast states of the country, IBGE estimates for adult mortality are much higher than any of the other studies in the analysis (Fig. 3). The difference is striking for some states such as Maranhão where IBGE estimates adult mortality around 0.293 for males compared to 0.176 from direct estimates (without correction), 0.183 from IHME, 0.193 from DDM-R and 0.22 from Bayesian. The last estimate also presents a very large uncertainty interval. On the other hand, for states in the South and Southeast, where completeness of death reporting is close to 100%, IBGE estimates show adult mortality to be lower than direct computations from MIS. DDM-R shows estimates a little higher than those from IHME and Bayesian but with relatively small differences and very close to what is obtained directly from the data.

**Fig. 4:**
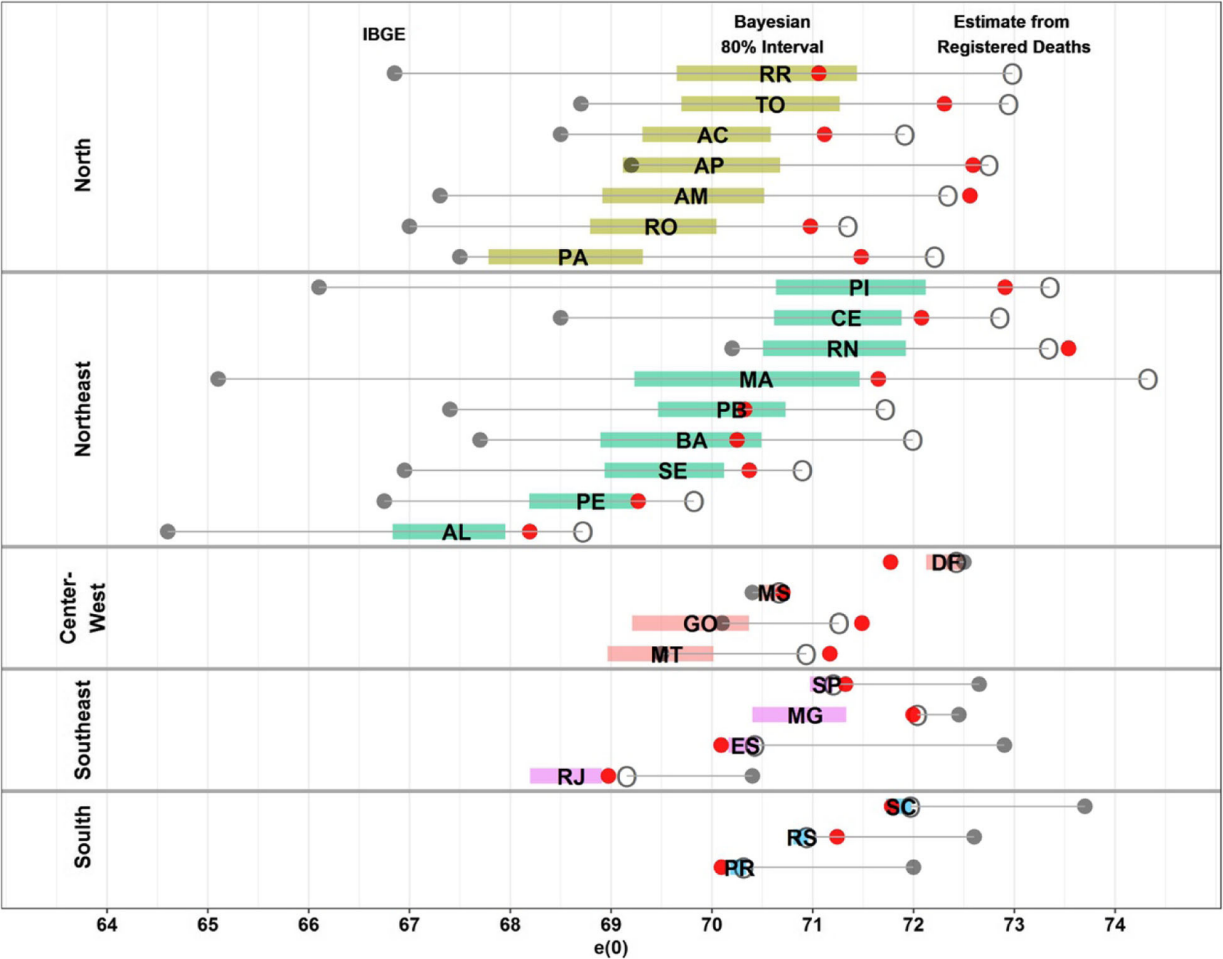
Males life expectancy at birth, 2010—Bayesian model, IHME, IBGE and MIS (Ministry of Health). Source: Schmertmann and Gonzaga [6], IHME [11] and IBGE [12]. Note: Shaded bars in different colours represent 80% posterior probability interval for life expectancy at birth—e(0)—from the Bayesian model; state abbreviations appear at posterior median; red solid dot represent IHME estimates, grey solid dot represent IBGE estimates and open circles represent unadjusted estimates from deaths registered by the Mortality Information System (MIS)

Figure 4 shows a summary of estimates of male life expectancy at birth in 2010 from different sources for Brazil and states. The effect of under-registration of deaths is clear when looking at estimates from direct data from MIS, which has not been corrected for under-reporting and these give the highest life expectancy for all states, mostly in the North and Northeast regions of the country. States of regions with better data quality have life expectancy estimates very similar to those obtained using observed data. The second most striking result is the comparison of IBGE with IHME and the Bayesian model. They are much lower in the states of North and Northeast regions and much higher in the South and Southeast reflecting the procedures to adjust completeness of death registration as discussed before.

Strategies and methods used to estimate the completeness of deaths and mortality rates for younger and older ages impact the estimates of life expectancy at birth. Schmertmann and Gonzaga [6] combine a relational model for mortality schedules with probabilistic prior information on completeness of death registration obtained from several studies, and from field audits done by public health experts [26]. In general, IHME estimates are more conservative in all North and Northeast states and in some Southern and Southeast states and closer to Bayesian estimates then IBGE ones. In most states, results from Bayesian model represent a balance between IBGE and IHME estimates. If we compare all estimates without any correction (open circle), we see that official life expectancy estimates (IBGE ones) for Southern and Southeastern states are implausibly high, even higher than life expectancy estimates using direct data from MIS. As pointed out by Schmertmann and Gonzaga [6], this indicates that the IBGE estimates are plausible only if the vital registration system substantially over-records deaths in these states.

One possible explanation of that overestimation of IBGE’s life expectancy in Southern and Southeastern states is related to its top-down estimation procedure. Since completeness of deaths estimate for each state and Brazil is estimated independently, the sum of estimated deaths across states can be greater than the total estimates of deaths for the country. Then, a top-down estimation procedure is applied in order to make the sum of deaths between states equal to national estimates. However, this procedure resulted in higher life expectancy at older ages in North and Northeast regions, compared to South and Southeast regions, which might be implausible since living conditions and health care system for the older population are better found in the South and Southeast regions [12]. Then, the IBGE solution was to exclude North and Northeast states of the top-down procedure. Therefore, the excess of estimated deaths across states was eliminated only in South and Southeast states leading to a higher life expectancy in those states.

Lastly, important issues arise when one studies the single-age mortality profiles in both developed and developing countries. Data limitation at older ages could explain the differences in ranking of e(0) from different methods (see Fig. 4). Feehan [28] shows that mortality estimates at older ages, beyond age 80, are limited because both exposure and events are rare. In the case of Latin America and Brazil, the issue might be aggravated due to errors in age reporting [29]. In the construction of the Latin America Mortality Database, they use the mortality age profile of Costa Rica, considered to be adequate, to adjust older age mortality for other countries. However, a recent analysis argues that adult and old-age mortality, in Costa Rica, are unexpectedly low compared to the levels of infant and child mortality across different regions [30]. Comparisons of the schedule of mortality rates at older ages between states in 2010 highlight that age miss-reporting in both deaths and population, combined with under-recording of deaths by the vital registration system, lead to a crossover of mortality rates at older ages between South and North/Northeast states. Similar evidence was observed in several studies, using Brazilian data, for the centenarian population indicating that the observed number of individuals above age 100 is heavily affected by age misstatements [31, 32].

**Fig. 5:**
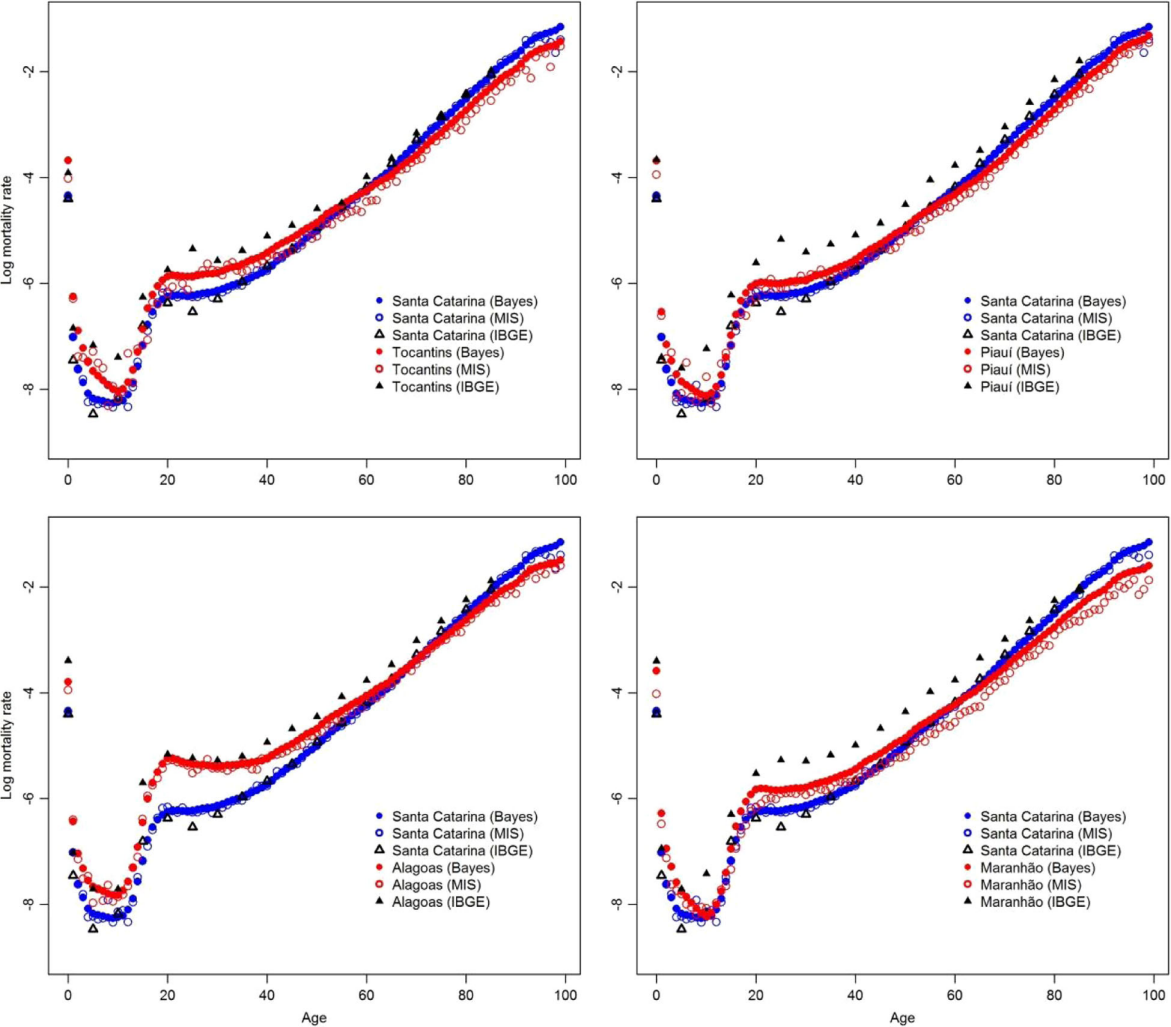
Males log mortality rates by single ages from Bayesian model and mortality information system and by 5 years age interval from IBGE, for selected states in Brazil in 2010. Source: Schmertmann and Gonzaga [6] and IBGE [12]

Figure 5 shows the observed and smoothed males’ mortality rates by single ages for five selected states in 2010, two states in North regions (Tocantins and Piauí), two states in Northeast region (Alagoas and Maranhão) and Santa Catarina (located in the South region). The smoothed male mortality rates by age (solid dot) come from the posterior distribution for mortality rates according to the Bayesian model [6]. The observed rates by single ages (open circle) come from vital registration system without any correction. Open and solid triangles come from IBGE estimates for 5-year age intervals^2^. Comparisons of males’ age-specific mortality rates between Santa Catarina and the North/Northeast states show a clear crossover of mortality rates starting around 50 or 60 years old. The crossover does not disappear even after corrections due to undercounting of deaths by age based on Bayesian model results, even in Maranhão state where the adjustment due to undercount of deaths is more evident.

The crossover of mortality rates at advanced ages between South and North/Northeast states has at least three possible causes. First, it could be possible that the completeness of deaths at advanced ages in North/Northeast states are lower than at adult and young ages [28, 33]. Second, it could be a consequence of different age misreporting patterns between South and North/Northeast states in line with what was observed in Nepomuceno and Turra [31] and Gomes and Turra [32] for the centenarian population. Preston et al. [34] investigated how different types of age misreporting can affect estimates of mortality rates at older ages, by analysing the effects of three patterns of age misreporting: net age overstatement, net age understatement and symmetric age misreporting. Based on those three types of age misreporting and on five types of mortality estimates, they found that age misstatement biases mortality estimates underestimated at advanced ages. The third possible cause is not related to data quality but to mortality selection [35]. In the context of higher mortality rates at young ages, survivors to older ages would be physiologically stronger and then live longer than others. Finally, it could be possible that the crossover of mortality rates between South and North/Northeast states arises from different data problems and from mortality selection.

## Discussion

Mortality estimates are central to the Global Burden of Disease analysis—called “mortality envelope”. In countries with a long-time series and good data quality, mortality estimates are more stable and require fewer adjustments. Estimates of adult mortality remain a challenge for demographers and public health researchers in most less developed countries, and one of those challenges is to overcome the lack of quality of vital data. In countries, such as Brazil and its states, vital registration systems are recent and are still subject to many limitations [3, 4, 36]. Thus, it is not a straightforward exercise to obtain estimates on the levels and trends of mortality. It is necessary to perform a series of adjustments before having adequate levels and trends of mortality in the country and states.

We compared the IHME, IBGE, DDM-R and Bayesian Model estimates of adult mortality (_45_q_15_) and life expectancy at birth from 1980 to 2010 at the subnational level. We find that the estimates for all the authors are very similar at the country level, confirming that Brazil has made significant progress in improving the quality of mortality data over the last three decades. However, differences between the four sources of estimates of completeness and mortality are presented at state level and throughout the whole period of analysis.

The importance of this issue is increased by the fact that the recent version of the Brazil Burden of Disease includes estimates at the subnational level and there are plans to go to even smaller areas. Small area estimates face additional problems owing to low levels of completeness: fluctuations due to small numbers of events and random variation. In general, demographic methods have a number of limitations when applied to estimate concerning aforementioned areas, and in recent years, several studies tried to incorporate statistical models to obtain more robust estimates of mortality and life expectancy [6, 7, 37]. Since we do not know the true level of mortality and there is a range of estimates in Brazil, it is important to compare and contrast these estimates.

Our analysis shows that data quality has improved in recent decades but the quality of data are still deficient for many states, mostly the less developed ones [13]. The possible causes for an imprecise vital record could be many, and what is more, this lack of data quality could compromise even the most precise and robust estimates of mortality. Our results are very similar to what Schmertmann and Gonzaga [6] observed when comparing their estimates to IBGE. But, we find some differences in the estimates of IHME and Brazilian researchers that affect both levels and trends of completeness of adult mortality in Brazil and states, although there is a clear convergence trend in the most recent period.

Substantial differences between estimates may exist because of differences in data source, methods and/or modelling assumptions used. Then, it is very important that the data and methodology used by different researches be clear and reproducible by others. In most cases, because the methodology in papers and the results on websites are not very clear and methods and data are not available, it is not easy to reproduce results.

We should continuously incorporate new estimates in mortality analysis. Brazilian subnational mortality estimates represent an important step for mortality studies in less developed countries with limited data. The next step would be to analyse mortality estimates for those aged 60 and over because a good part of cross-over in mortality between states may be in the data quality at older ages. Furthermore, it would be an important gain for mortality studies to produce estimates with confidence intervals.

There are other methodological alternatives to exploit the quality of mortality data and produce estimates of adult mortality and life expectancy at birth. The Preston Integrated Method [38] provides an alternative way to estimate a life table from two census-age distributions. This approach requires, in addition to two age distributions, a rough estimate of the probability of survival of the population from birth to age *x*, ps (*x*), function (using a logit transformation of a parameter) and, if possible, an estimate independent of the probability of surviving at age 5 years, p (5). The immediate question, however, is what to use as the standard survival function ps (*x*), which should represent the true age pattern of mortality as much as possible. In a population with death registration that is 80% or more complete, one possibility is to use a life table derived from recorded deaths and population; if the death register does not vary greatly by age, the resulting survival function will not be too distorted in the age pattern. An alternative suggested by Preston [38] is to use a model life table with a life expectancy close to what is believed to be appropriate for the population in question.

Adair and Lopez [39] suggest an empirical model to generate estimates of under-registration correction with applicability for small areas. The method demonstrates sufficient flexibility to predict a wide range of completeness levels at a given gross registered mortality rate. The method can be applied using data readily available at the subnational level. The model assumes that the degree of coverage of the observed death as a positive relation with the crude mortality rate recorded, a negative relation with the level of mortality and a negative relation with the older age structure of the population. The model was adjusted for data from more than 100 countries, and the estimated parameters can be used to obtain estimates of the degree of coverage in other countries or localities around the world One major limitation is that it is calibrated on IHME estimates and completeness can never exceed 100%. Schmertmann and Gonzaga [6] proposed the combination of demographic methods, rate smoothing based on a relational model together with the use of Bayesian statistics to obtain estimates of data quality and thus corrected mortality curves for small areas. The proposed model combines a relational model for mortality curves with probabilistic prior information on death record coverage derived from demographic estimation techniques such as death distribution methods and field surveys by public health experts.

The comparison between the estimates of completeness of death registration, life expectancy and adult mortality represents a useful strategy to evaluate the potential of each method. We find substantial sub-national differences between estimates of completeness of death registration, adult mortality and life expectancy between sources analysed in this paper. The issues exist because of various differences in data and modelling assumptions used by each agency and author. It is possible that those differences reflect the limitations of data and methods, but the differences should be reduced as data quality improves. In addition, better transparency on methods and data used will help to improve understanding about the drivers of the differences. We expect that the methods should be widely and easily applied, with the purpose of providing reliable mortality statistics for the public policy planning.

## Conclusion

We have showed that the quality of mortality data in Brazil has improved steadily overtime, but with large regional variations. However, we observed that IBGE estimates show the lowest levels of completeness for the Northern part of the country compared to other estimates. Choice of methods and approaches might lead to very unexpected results and conclusions. We have produced a detailed comparative analysis of estimates of completeness of death registration by different sources and have discussed the main results and possible explanations for these variations. We have made it clear that new improved methods are still needed in order to study adult mortality in less developed countries and at the subnational level. More comparative studies are necessary for improvement of quality of mortality estimates in Brazil.

## Supplementary information

**Additional file 1:** **Table S1.** Estimates of Completeness of Death Counts Coverage, Brazil, 1980/1991 and 2000/2010. Source: Queiroz, et.al [9], I.H.M.E [11], I.B.G.E [12].

## Data Availability

The datasets generated and/or analysed during the current study are public available at: a) Institute of Health Metrics and Evaluation (IHME) http://www.healthdata.org/ b) Instituto Brasileiro de Geografia e Estatística (IBGE): www.ibge.gov.br c) Schmertmann, C. P., & Gonzaga, M. R. (2018). Bayesian estimation of age-specific mortality and life expectancy for small areas with defective vital records. Demography, 55(4), 1363-1388. http://mortality-subregistration.schmert.net/ d) Queiroz, B. L. et al. Completeness of death-count coverage and adult mortality (45q15) for Brazilian states from 1980 to 2010. Revista Brasileira de Epidemiologia, v. 20, n. supl 1, p. 21-33, 2017.

## Abbreviations

DDM: Death distribution methods
GBD: Global Burden of Disease
GGB: General growth balance
IBGE: Instituto Brasileiro de Geografia e Estatistica
IHME: Institute of Health Metrics and Evaluation
MIS: Ministério da Saúde/Ministry of Health
SEG: Synthetic extinct generations

## Footnotes

1 More details about the IBGE to produce their life table estimates in 2010 are available at www2.ibge.gov.br/home/estatistica/populacao/tabuadevida/metodologia.shtm.

2 IBGE did not published UF estimates of mortality probabilities by single years of age.
