# Supplementary material for: Comparative analysis of completeness of death registration, adult mortality and life expectancy at birth in Brazil at the subnational level
Source: Popul Health Metr. 2020 Sep 30;18(Suppl 1):11. doi: 10.1186/s12963-020-00213-4 (PMC7525963; doi:10.1186/s12963-020-00213-4)
Supplement: Supplementary file 1 — Additional file 1: Table S1. Estimates of Completeness of Death Counts Coverage, Brazil, 1980/1991 and 2000/2010. Source: Queiroz, et.al [9], I.H.M.E [11], I.B.G.E [12]. [file 12963_2020_213_MOESM1_ESM.docx]

|  |  |  |  |  |  |  |
| --- | --- | --- | --- | --- | --- | --- |
| **State and Region** | **Queiroz 80/91** | **IHME 80/91** | **IBGE 80/91** | **IHME 00/10** | **IBGE 00/10** | **Queiroz 00/10** |
| **NORTHEAST** |  |  |  |  |  |  |
| AL | 0,989 | 1,000 | 0,713 | 1,000 | 0,752 | 0,950 |
| PE | 0,898 | 0,798 | 0,508 | 0,917 | 0,548 | 0,855 |
| SE | 1,023 | 1,000 | 0,962 | 1,000 | 0,995 | 0,994 |
| BA | 0,938 | 0,959 | 0,630 | 0,894 | 0,686 | 0,901 |
| PB | 0,957 | 1,000 | 0,557 | 1,000 | 0,721 | 0,835 |
| CE | 0,747 | 0,724 | 0,580 | 0,943 | 0,601 | 0,874 |
| RN | 0,909 | 0,940 | 0,698 | 1,000 | 0,812 | 0,931 |
| MA | 0,917 | 0,857 | 0,726 | 0,939 | 0,757 | 0,914 |
| PI | 1,136 | 1,000 | 0,951 | 1,000 | 1,000 | 1,008 |
| **NORTH** |  |  |  |  |  |  |
| RO | 1,004 | 1,000 | 0,993 | 1,000 | 1,000 | 1,000 |
| AC | 0,957 | 1,000 | 0,729 | 0,991 | 0,635 | 0,921 |
| PA | 0,507 | 0,623 | 0,619 | 0,911 | 0,655 | 0,852 |
| AM | 0,980 | 1,000 | 0,693 | 1,000 | 0,688 | 0,933 |
| TO | 0,992 | 1,000 | 0,874 | 1,000 | 0,963 | 0,936 |
| AP | 0,831 | 0,895 | 0,646 | 0,902 | 0,720 | 0,889 |
| RR | 1,062 | 1,000 | 0,984 | 1,000 | 0,994 | 0,960 |
| **MID-WEST** |  |  |  |  |  |  |
| DF | 0,931 | 0,855 | 0,537 | 0,960 | 0,615 | 0,889 |
| GO | 0,583 | 0,693 | 0,402 | 0,918 | 0,469 | 0,871 |
| MS | 0,821 | 0,936 | 0,628 | 0,943 | 0,622 | 0,770 |
| MT | 0,779 | 0,918 | 0,619 | 1,000 | 0,645 | 0,910 |
| **SOUTHEAST** |  |  |  |  |  |  |
| SP | 1,292 | 1,000 | 0,903 | 1,000 | 0,951 | 0,994 |
| RJ | 0,965 | 1,000 | 0,763 | 1,000 | 0,889 | 0,972 |
| MG | 1,178 | 1,000 | 0,681 | 1,000 | 0,789 | 0,868 |
| ES | 0,574 | 0,608 | 0,415 | 0,799 | 0,401 | 0,723 |
| **SOUTH** |  |  |  |  |  |  |
| RS | 1,009 | 1,000 | 0,908 | 1,000 | 0,922 | 0,930 |
| PR | 0,912 | 1,000 | 0,817 | 1,000 | 0,859 | 0,912 |
| SC | 0,952 | 1,000 | 0,834 | 1,000 | 0,947 | 0,990 |
|  |  |  |  |  |  |  |
